# Symptomatic Dengue during Pregnancy and Congenital Neurologic Malformations

**DOI:** 10.3201/eid2409.170361

**Published:** 2018-09

**Authors:** Enny S. Paixão, Maria Glória Teixeira, Maria da Conceição N. Costa, Mauricio L. Barreto, Laura C. Rodrigues

**Affiliations:** London School of Hygiene and Tropical Medicine, London, United Kingdom (E.S. Paixão, L.C. Rodrigues);; Instituto de Saúde Coletiva, Salvador-Bahia, Brazil (E.S. Paixão, M.G. Teixeira, M. da Conceição N. Costa, M.L. Barreto);; Center of Data and Knowledge Integration for Health (CIDACS), Salvador-Bahia (M.L. Barreto, L.C. Rodrigues)

**Keywords:** dengue, birth defects, neurologic malformations, congenital anomalies, pregnancy, viruses, vector-borne infections, dengue virus

## Abstract

Dengue virus infection during pregnancy increased the risk for any neurologic congenital anomaly in the infant by roughly 50% and for other congenital malformations of brain 4-fold. Our results show an association between dengue during pregnancy and congenital anomalies of the brain, suggesting that flaviviruses other than Zika virus are associated with such malformations.

Before the causal relationship between Zika virus and neurologic congenital anomalies (*1*), especially microcephaly (*2*), was established, no evidence associated flavivirus with congenital malformations in humans, although postnatal complications have been described (*3*). We investigated whether dengue virus (DENV) infection during pregnancy could be associated with neurologic defects in the infant at birth.

We conducted a population-based study using routinely collected data from live births and from women who were notified and confirmed to have DENV infection during 2006–2012 in Brazil, before the introduction of Zika virus. We probabilistically linked records of mothers of live births with records of dengue notification to identify women who were reported as having dengue during pregnancy. We excluded records with missing or implausible names, multiple pregnancies, and births in municipalities with no dengue notifications. We obtained ethics approval from Federal University of Bahia, Salvador, Brazil (CAAE: 26797814.7.0000.5030) and from London School of Hygiene and Tropical Medicine (Ethics Ref:10269).

In the matching process, we used name, age, and place of residence of the mother at time of delivery and notification. We included only links and nonlinks with a high degree of certainty. We validated the linkage process in a study that demonstrated 62% sensitivity (*4*). 

We used an outcome definition of congenital malformation of the nervous system coded as Q00-Q07 in International Classification of Diseases, 10th Revision (ICD-10). We defined dengue as a confirmed case of DENV infection notified during a pregnancy that resulted in a live birth. We estimated the association between symptomatic dengue during pregnancy and neurologic congenital malformations using the Firth method to reduce the small sample bias in maximum-likelihood estimation.

The study parameters encompassed 16,103,312 live births. Neurologic congenital anomalies are rare; they occurred in 13,634 (0.08%) live births. Dengue during pregnancy increased the odds of a neurologic congenital anomaly by 50% (Table), but this result was not statistically significant (95% CI 0.97–2.27). We split the neurologic congenital defects into ICD-10 categories; the 95% CI around the estimated odds ratios (ORs) was not statistically significant in 7 categories, including microcephaly (OR 1.7, 95% CI 0.33–8.32). Two other types of neurologic congenital anomalies were >4 times more frequent in women who had DENV infection during pregnancy: other congenital malformations of spinal cord (OR 5.4, 95% CI 1.0–26.9) and other congenital malformations of brain (OR 4.5, 95% CI 1.7–11.3, which was statistically significant). We found no sign of space-time clusters or recording errors suggestive of a coding artifact in the 4 records of other congenital malformations of brain wherein the mother had DENV infection (Technical Appendix Table)

**Table:** Association between congenital anomalies and symptomatic dengue virus infection during pregnancy, Brazil, 2006–2012

Congenital anomaly	Odds ratio (95% CI)
Neurologic congenital anomalies	1.5 (0.9–2.2)
Anencephaly	1.9 (0.8–4.4)
Encephalocele	1.4 (0.3–6.9)
Microcephaly	1.7 (0.3–8.3)
Congenital hydrocephalus	1.6 (0.8–3.2)
Other congenital malformations of brain	4.5 (1.7–11.3
Spina bifida	0.8
Other congenital malformations of spinal cord	5.4
Other congenital malformations of nervous system	Not available

Symptoms of DENV infection occurred in the first trimester in 50% of patients. The specific diagnosis of those among the nonexposed group were congenital malformation of corpus callosum (9%; 81/943), holoprosencephaly (24%; 225/943), and septooptic dysplasia (0.6%; 6/943).

Our study showed an association between DENV infection during pregnancy and congenital anomalies of the brain. Congenital anomalies of the brain detectable by routine examination at birth are so rare (6/100,000 live births by our data) that it was necessary to assemble a cohort of >16 million live births to detect an effect of dengue; even then, we did not have sufficient power to confidently exclude associations with other neurologic abnormalities. 

Because DENV infection had not been associated with congenital anomalies, there is no established biologic mechanism for its teratogenicity. However, there is evidence for postnatal neurotropism and virus isolation from brain tissue (*5*) and for dengue virus crossing the blood–brain and placental barriers (*6*,*7*). The pattern of anomalies we described has similarities with congenital Zika syndrome. Brain images and autopsies from infants with Zika and other infectious diseases have revealed abnormalities similar to those we described (*8*,*9*). 

Our study has limitations inherent to the linkage process. Rigorous evaluation of the linkage process showed that it is unlikely to introduce bias and that it did not affect the magnitude of the association (*4*). Another potential limitation was diagnosis of DENV infection. In notifiable epidemics, not all cases are tested after the cause is established. DENV infection in Brazil is notified for the presence of clinical criteria, laboratory confirmation, or both. Only ≈30% of notified DENV infections are laboratory confirmed, which could lead to bias if unconfirmed cases are not dengue. However, a previous article found no difference in pregnancy outcomes for women with notified DENV infection with and without laboratory confirmation (*10*). We did not control for potential confounders, so confounders such as maternal illness or environmental exposures may have contributed to the association between dengue infection and neurologic malformations.

The association of symptomatic dengue during pregnancy and congenital anomalies of the brain in the infant, while not as high frequency as the linkage with Zika, opens the possibility of other flaviviruses causing congenital malformations and raises questions about policy implications. We recommend careful observation and recording of DENV infection in antenatal records and full investigation of live births with neurologic malformations, as well as animal and in vitro research of teratogenic effects of dengue.

Technical Appendix Additional information about the association between dengue virus infection during pregnancy and other congenital malformations of brain.
