# Supplementary material for: Symptomatic Dengue during Pregnancy and Congenital Neurologic Malformations
Source: Emerg Infect Dis. 2018 Sep;24(9):1748–50. doi: 10.3201/eid2409.170361 (PMC6106414; doi:10.3201/eid2409.170361)
Supplement: Technical Appendix — Additional information about the association between dengue virus infection during pregnancy and other congenital malformations of brain. [file 17-0361-Techapp-s1.pdf]

Article DOI: https://doi.org/10.3201/eid2409.170361

Symptomatic Dengue during Pregnancy and Congenital Neurologic Malformations

Technical Appendix

Technical Appendix Table. Descriptions of 4 cases of dengue virus exposure during pregnancy associated with other congenital malformations of brain in the infant

Municipality	Date of birth	Mother's age, y	Mother's education level, y	Anomaly	Trimester of dengue exposure	Gestational age, wk	Birth weight, g
Maracanau, CE	05/2008	22	8–11	Congenital malformation of brain, unspecified	3	32–36	2,000
Belo Horizonte, MG	10/2008	24	8–11	Corpus callosum	1	37–41	3,570
Campinas, SP	11/2010	28	4–7	Holoprosencephaly	1	37–41	1,855
Fortaleza, CE	09/2011	35	8–11	Septo-optic dysplasia	2	37–41	1,760
